# COMT inhibition with entacapone for patients with Parkinson’s disease and motor complications: the novelty of continuous infusion

**DOI:** 10.1007/s00702-025-03006-x

**Published:** 2025-09-02

**Authors:** Peter Jenner, Dag Nyholm

**Affiliations:** 1 Institute of Pharmaceutical Sciences, Faculty of Life Sciences and Medicine, King’s College London, London, SE1 1UL UK; 2 Department of Medical Sciences, Neurology, Uppsala University, Uppsala, Sweden

**Keywords:** COMT inhibition, Entacapone, Infusion, Levodopa, Motor fluctuations, Parkinson’s disease

## Abstract

Enhancing levodopa efficacy through peripheral COMT inhibition and using continuous infusion are both established strategies for managing motor fluctuations. Both approaches aim to improve the pharmacokinetics of levodopa in order to maintain a steady delivery of levodopa to the brain, avoid the deep troughs in levodopa plasma concentrations associated with oral levodopa delivery, and thereby reduce OFF time. In this review, we describe the pharmacologic rationale for combining COMT inhibition with entacapone and continuous dopaminergic delivery that led to the development of levodopa/entacapone/carbidopa intestinal gel (LECIG). Entacapone is a peripheral COMT inhibitor, that effectively reduces the metabolism of levodopa to 3-O-methyldopa and increases the amount of levodopa that can be transported into the brain. However, entacapone has a short half-life and poor oral bioavailability that necessitates frequent dosing for optimal efficacy. Continuous infusion of entacapone allows for improved bioavailability from direct delivery to the duodenum/jejunum. As such, lower overall levodopa doses can be given to achieve therapeutically effective concentrations. Accumulating real-world evidence with LECIG supports its use in managing motor fluctuations in patients whose control is becoming suboptimal with oral approaches. We provide an overview of the growing evidence base for its risk-benefit profile in patients with motor fluctuations.

## Introduction

Parkinson’s disease (PD) is the fastest growing neurological disorder worldwide and the number of people with PD (PwP) is predicted to grow as a consequence of both population aging and increased life expectancy improvement (Dorsey and Bloem 2018; Wanneveich et al. 2018). By 2030, a person diagnosed with PD is expected to live 16–18 years with the disease (Wanneveich et al. 2018). However, with disease progression, the clinical management of PD typically becomes increasingly complex (Titova et al. 2017) and it is expected that the prolonged life expectancy will significantly increase the proportion of patients living with advanced PD.

**Fig. 1:**
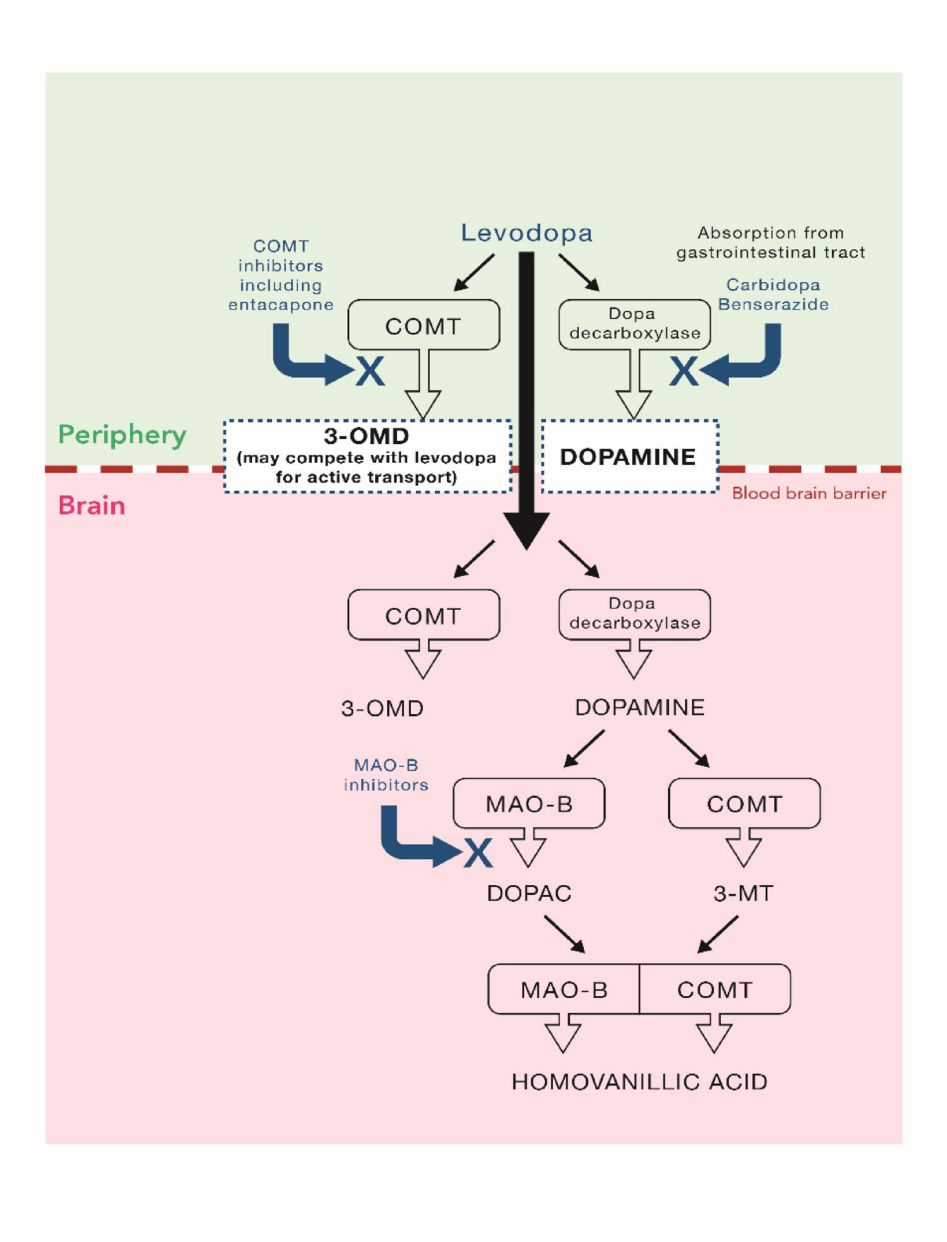
Levodopa and dopamine metabolic pathways

The mainstay of PD pharmacotherapy is levodopa combined with a dopa decarboxylase inhibitor (DDCi; namely carbidopa or benserazide) that acts to block a major pathway of peripheral metabolism and improve brain penetration and levodopa’s overall bioavailability (Olanow 2019). The common use of immediate release oral levodopa/DDCi reflects its efficacy and safety and virtually every PwP will begin its use in the early to moderate phases of the disease. However, with long term use, therapy becomes complicated by the development of motor fluctuations and dyskinesia (Gandhi et al. 2024; Kim et al. 2020). These are related, in part, to levodopa’s short plasma/brain half-life (Bastide et al. 2015) and necessitate increasingly frequent oral administration (Nyholm and Stepien 2014) or the introduction of long acting forms of the drug to increase availability to the brain and increase ON time. When motor fluctuations develop, levodopa is commonly given with oral adjunct therapies such as MAO-B inhibitors (selegiline, rasagiline, safinamide) which act to prolong the duration of effect of dopamine formed from levodopa in the striatum by blocking dopamine’s metabolism so reducing OFF time by improving ON periods. However, MAO-B inhibitors do not improve the brain delivery of levodopa and a more holistic approach is to use the catechol-*O*-methyltransferase (COMT) inhibitor entacapone to block the other major pathway of levodopa’s peripheral metabolism, improve its bioavailability, increase brain penetration and extend clinical efficacy (Müller 2013). The use of the three classes of enzyme inhibitors (DDC, MAOB, and COMT inhibitors, Fig. 1) as adjuncts to levodopa have all been demonstrated to produce clinically relevant improvements in the control of motor function in PD.

With disease progression, however, the oral approach often becomes insufficient for symptomatic control (Deuschl et al. 2022) and continuous levodopa infusion therapies have been developed for patients with more advanced motor complications (Olanow et al. 2014; Soileau et al. 2022; Espay et al. 2024). While pilot studies of intravenous levodopa infusions provided proof of concept (Hardie et al. 1984; Quinn et al. 1982), the poor solubility of levodopa led to the development of microsuspension formulations that can be delivered directly to the jejunum (Aquilonius and Nyholm 2017). A highly concentrated formulation of the pro-drugs fos-levodopa/fos-carbidopa has also become available for subcutaneous delivery (Soileau et al. 2022). These advanced therapies still require the use of a DDCi, invariably carbidopa, as the extensive peripheral metabolism of levodopa would otherwise negate the benefits of continuous drug delivery. By contrast, the use of COMT inhibitors to further reduce levodopa’s peripheral metabolism and increase its bioavailability to brain has not been routinely incorporated into these continuous treatment regimens despite evidence that COMT inhibition improves the plasma pharmacokinetic profile of infused levodopa and enhances efficacy (Buhmann et al. 2017; Antonini et al. 2017).

Recently, a novel levodopa/entacapone/carbidopa intestinal gel (LECIG, commercialised as LECIGON^®^, STADA) has been introduced for continuous intraduodenal administration in fluctuating PD (Nyholm et al. 2012; Senek et al. 2017). This review looks at the rationale for combining COMT inhibition and continuous levodopa infusion and reviews the benefit that this combination of approaches brings in terms of improved efficacy and reduced adverse event profile.

## The physiologic role of catechol-*O*-methyltransferase (COMT)

As an introduction to the use of COMT inhibitors in PD, some basic information of COMT needs to be covered. First isolated and purified by Axelrod and Tomchick in 1958, COMT is a ubiquitous enzyme that catalyses the metabolic inactivation of endogenous catechols and xenobiotics with a catechol structure by *O-*methylation (Guldberg and Marsden 1975). Methylation takes place in the presence of S-adenosyl-methionine with magnesium (Mg^2+^) as a cofactor and usually results in the complete loss of the pharmacological activity of the substrate molecule. Typical endogenous substrates include catecholamine neurotransmitters dopamine, noradrenaline, and adrenaline (Nissinen and Männistö 2010). Its role in the metabolism of levodopa was discovered early on, and it was quickly realised that levodopa’s major metabolites are 3-O-methyldopa (3-OMD) and homovanillic acid (HVA)/ 3-methoxytyramine respectively (Hornykiewicz 2010). In the case of 3-OMD, O-methylation results in a structure that is no longer a substrate for dopa decarboxylase and as a consequence 3-OMD accumulates in tissues on prolonged levodopa administration (Nissinen 2010).

COMT activity can be detected in virtually all organs and brain areas (Myöhänen and Männistö 2010). COMT exists as two isoforms – membrane bound COMT and soluble COMT (primarily localised in the cytoplasm and nucleus). The membrane-bound form (MB-COMT) predominates in the central nervous system and is believed to be involved in acute immune defence (as supported by high levels of expression in microglia in response to inflammation (Reenilä et al. 1997) and the metabolism of endogenous catecholamines, including levodopa and dopamine, at physiological concentrations (Roth 1992). Interestingly, and perhaps surprisingly, levels of COMT activity are low in the dopaminergic nigrostriatal pathway (Myöhänen and Männistö 2010). However, COMT activity is higher in the peripheral tissues than in the CNS and the soluble cytosolic form (S-COMT) predominates, with the highest levels of activity in the liver, kidney, and gastrointestinal tract (Myöhänen and Männistö 2010). Peripheral S-COMT has been referred to as an ‘enzymatic detoxifying barrier’ between the blood and other tissues (Kaakkola et al. 1994); it has a low affinity for catecholamine substrates but very high capacity and is thought to be mainly responsible for the elimination of exogenously delivered catechols, including levodopa. Accordingly, it is the most relevant form to target when delivering levodopa as a dopamine replacement therapy in PD.

Of note, certain single-nucleotide polymorphisms (SNPs) in the human COMT gene (located on chromosome 22q11.21) have been suggested to play a role in the susceptibility to PD (Bialecka et al. 2012). COMT polymorphisms affect dopamine clearance and polymorphisms – most commonly the Val158Met variant – are associated with high (Val-Val), intermediate (Val-Met) and low (Met/Met) levels of enzyme activity. Patients with the Met/Met variant have been reported to have a lower risk of motor fluctuations (Wu et al. 2014), while patients with the Val/Val variant are reported to have a better response to COMT inhibitors, including entacapone (Corvol et al. 2011).

## Utilising COMT inhibition to optimise the peripheral pharmacokinetics of Levodopa

The peripheral metabolism of levodopa is accepted as being key to optimising levodopa delivery to the brain for the effective symptomatic management of PD. The clear advantages of using a DDC inhibitor with levodopa are widely understood to the point where this is standard clinical practice in both early and late-stage PD. COMT also has an established role in the peripheral metabolism of levodopa and COMT inhibitors have been used accordingly. But what is less well appreciated is that when levodopa is given with a DDC inhibitor, its metabolism promptly shifts to the COMT pathway where it is rapidly inactivated by methylation to 3-O-methyldopa (3-OMD) (Nissinen and Männistö 2010) (Fig. 1). This serves to reduce levodopa’s bioavailability such that only 5–10% of levodopa given with a DDC inhibitor reaches the brain (Kaakkola 2000). In addition, 3-OMD (with a half-life of > 10 h) also accumulates in the brain and periphery and may compete with levodopa for active transport into the brain (Calne et al. 1973), making it even more important to ensure that both DDC and COMT remain functionally inhibited during levodopa therapy.

3-OMD has also been implicated in the development of motor complications (Tohgi et al. 1991) as well as hyperhomocysteinaemia, which may be an important factor in the development of peripheral neuropathy (Müller et al. 2013). The prevalence of peripheral neuropathy is increased in PD compared to the age-matched general population and may be related to levodopa intake and/or peripheral alpha-synuclein aggregation (Zis et al. 2017). Importantly, COMT-mediated conversion of levodopa into 3-OMD depletes methyl-group reserves and leads to homocysteine production and depletion of vitamins B6, B12, and folate (Romagnolo et al. 2019; Ahlskog 2023). The latter being important because B6 supports the conversion of homocysteine to cysteine and B12 and folate support the metabolism (re-methylation) of homocysteine to methionine. Inhibition of COMT may therefore reduce the consumption of B vitamins that is otherwise related to levodopa therapy.

## COMT inhibition in PD—the role of Entacapone

While the importance of COMT in the metabolism of levodopa was recognised early, attempts at producing a COMT inhibitor for use in PD were unsuccessful. Compounds such as gallates and tropolone were investigated but had low potency, poor selectivity for COMT, were short acting and generally toxic. Only in the 1980’s with the development of potent, selective and orally active nitrocatechol compounds (‘the capones’) was use in PD made possible. Subsequently three ‘capones’ have been introduced into therapy for PD namely tolcapone, entacapone and opicapone. The capones vary in their spectrum of activity relative to the periphery and the brain. Tolcapone is currently the only COMT inhibitor to have both peripheral and central activity (Brannan et al. 1992) while both entacapone and opicapone selectively inhibit peripheral COMT activity and the soluble isoform of the enzyme. While central COMT inhibition will affect the metabolism of levodopa, the functional and clinical relevance of this to PD is not known. The most comprehensive experience of the use of COMT inhibition centres on entacapone use over several decades. Tolcapone use has been marred by the occurrence of rare cases of hepatotoxicity and despite additional safety studies, the drug has become black boxed and difficult to use on a routine basis because of the frequent liver function testing required. Opicapone is a newer addition to the COMT inhibitor family and remains to be fully utilised in PD.

The ability of the ‘capones’ to inhibit peripheral S-COMT activity and potentiate the delivery of levodopa to brain after oral administration was established in a range of preclinical models of PD where they each enhanced levodopa induced motor activity (Ettcheto et al. 2020; Männistö and Kaakkola 1999; Smith et al. 1997; Bonifacio et al. 2021). In man, the currently available COMT inhibitors consistently reduce OFF time and increase ON time in patients with PD experiencing motor complications when used as an adjunct to levodopa. A recent meta-analysis of oral COMT inhibitors as a class reported an average reduction in OFF time of about 0.8 hours accompanied by an average reduction in levodopa dose of 94 mg/day to balance out an increase in dyskinesia (Sisodia et al. 2024). Dopaminergic events, including dyskinesia and nausea, are among the most common events with adjunct COMT inhibition, and are related to the potentiated effects of levodopa.

Entacapone remains the most used COMT inhibitor. However, the oral use of entacapone has pharmacokinetic-pharmacodynamic deficiencies which are listed below and based on a review by Kaakkola in 2010:

1. Entacapone has a short elimination half-life when single oral doses are administered (estimated at 0.6 h) leading to fluctuations in plasma levels and difficulty in reaching steady state concentrations with standard intermittent oral administration (Rouru et al. 1999). This short half-life necessitates multiple doses per day, and oral entacapone is typically given with every levodopa dose.
2. Entacapone has a relatively low oral bioavailability of 35% (Habet 2022), compared to 60% for tolcapone (Jorga et al. 1998) and 20% for opicapone (Ongentys 50 mg 2024). Low oral bioavailability is associated with greater inter- and intra-individual variability in plasma concentrations and, consequently, in clinical response. For entacapone, this variability is evident with repeated dosing, as interindividual plasma levels after oral administration can differ by more than tenfold (Rouru et al. 1999).
3. Oral entacapone slows oral levodopa absorption and can inhibit levodopa and DDC inhibitor absorption (‘DDC leakage’) at higher doses (> 200 mg) prolonging the time to reach maximum plasma levels (Contin et al. 2008) Which may in turn delay the time-to-ON.
4. Oral administration of entacapone does not fully inhibit S-COMT activity. Single doses of entacapone 200 mg achieves around 60% peripheral COMT inhibition – potentially limiting the therapeutic effect.
5. After repeated oral administration of entacapone, COMT inhibition fluctuates over the course of the day and the inhibition is less than 10% after an over-night drug free period (Kaakkola 2010).

Thus, while combining conventional oral levodopa with adjunct oral entacapone helps avoid the low levodopa plasma trough levels that are directly associated with OFF-time (Stocchi et al. 2003), fluctuations in levodopa PK are still apparent and very frequent administration of entacapone with every dose of levodopa is required for a stable effect. However, dosing intervals of < 3 h lead to accumulation of levodopa during the day and, consequently, increased dyskinesias (Kuoppamäki et al. 2009; Stocchi et al. 2010). The short plasma half-life of oral levodopa coupled to the almost short half-life of entacapone meant that for optimal effectiveness the administration of the drugs needed to be time locked together. According to a recent systematic review, adding adjunct entacapone to a levodopa regimen is equivalent to multiplying the concomitant levodopa dose by 1.33 (i.e. adding entacapone to 100 mg levodopa is equivalent to giving 133 mg levodopa) (Jost et al. 2023). The frequency of oral administration of entacapone required was also an issue with respect to patient compliance, medication timing and pill burden. For this reason, a ‘triple combination’ of levodopa with carbidopa and entacapone was introduced to ensure optimised oral delivery (Stalevo^®^) in 2003 (Männistö et al. 2024). This formulation was incorporated into general use based on demonstrating bioequivalence to separate levodopa/carbidopa and entacapone (Hauser 2004). However, the underlying pharmacokinetic-pharmacodynamic deficiencies of the oral administration of entacapone remained until the concept of using continuous drug delivery and intraduodenal administration of levodopa/carbidopa/entacapone was developed.

## Continuous dopaminergic delivery (CDD) and the development of Levodopa infusion strategies

The role of levodopa’s short half-life in contributing to the development of motor complications is well established. Patients with advanced nigrostriatal degeneration who only take oral levodopa three or four times a day will demonstrate significant peaks and troughs in levodopa plasma levels that are (i) strongly correlated with motor response and (ii) are thought to cause downstream changes in basal ganglia transmission that lead to dyskinesia (Olanow et al. 2006, 2020). Added to this complexity, levodopa absorption only occurs from the upper gastro-intestinal tract and it is now understood that PD impacts gastrointestinal (GI) function, causing a variety of GI barriers to oral delivery and absorption that also directly impact its peripheral pharmacokinetics (Leta et al. 2023). Declining efficiency of gastric emptying from the stomach to deliver oral levodopa to its sole absorption site in the proximal small intestine (duodenum and jejunum) has been recognised as one of the consequences of chronic PD and is strongly correlated with delayed time-to-ON (Doi et al. 2012; Nyholm and Lennernäs 2008).

Better understanding of these concepts led to the idea of continuous dopaminergic delivery (CDD) whereby levodopa and other medications are delivered continuously, for example by infusion, to avoid the pharmacokinetic pulsatility associated with oral levodopa delivery and reduce motor complications (Pirtošek et al. 2023). While the terms are often used interchangeably, CDD differs from the concept of continuous dopaminergic stimulation (CDS) which describes the theoretical tonic stimulation of striatal neurons under *normal* (i.e. nonparkinsonian) conditions (Olanow et al. 2006). When treating an advanced PD patient with significant neurodegeneration, achieving CDD is considered a more realistic goal of therapy than CDS (Timpka et al. 2016; Jenner 2024).

Intravenous levodopa delivery was initially explored in the 1970s and demonstrated that a stable infusion rate improved its peripheral pharmacokinetics and reduced motor fluctuations (Quinn et al. 1982). However, intravenous levodopa infusion cannot be sustained beyond a week due to the risk of thrombosis. Following this, various percutaneous endoscopic gastrostomy (PEG) and portable duodenal infusion systems were developed and showed similarly stable levodopa plasma levels and clinical benefits to intravenous infusion (Sage et al. 1988; Kurth et al. 1993; Nyholm et al. 2003). The first levodopa infusion system to be commercialised was a levodopa/carbidopa enteral suspension delivered by a PEG-J (PEG with jejunal extension) (Aquilonius and Nyholm 2017). The efficacy and safety of levodopa-carbidopa intestinal gel (LCIG, commercialised as Duopa^®^/Duodopa^®^) has been established in several controlled clinical trials and in observational ‘real-world’ studies (Olanow et al. 2014; Antonini et al. 2017; Poewe et al. 2020; Fernandez et al. 2015; Freire-Alvarez et al. 2021; Chaudhuri et al. 2023; Nyholm et al. 2005). Under controlled conditions, the magnitude of benefit in reducing OFF time with continuous intestinal levodopa infusion [reduction of 1.9 h versus placebo (Olanow et al. 2014)] was considerably higher than what can be achieved with oral strategies [reductions of 0.8–1.1 h versus placebo (Sisodia et al. 2024)] and set a new bar for assessing the efficacy of pharmacotherapy in advanced disease. Sustained improvements in patients’ quality of life (Valldeoriola et al. 2021; Antonini et al. 2017) have also been reported. While AEs related to the PEG procedure or the infusion pump are known risks of intraintestinal infusion, they generally occur early on and are transient; patients are also generally well counselled and prepared for the surgical intervention (Antonini et al. 2017). A systematic review of the LCIG literature found that most discontinuations were due to device-related issues which occurred mostly within the first week and were usually resolved (Antonini et al. 2021). Consensus guidelines from Navigate PD note that patient frailty, meaning that the patient is unable to support the weight of the device, is an important consideration when considering LCIG (Odin et al. 2015).

LCIG is often described as a monotherapy (Buhmann et al. 2017) but real-world experience has shown that ≤ 40% of patients are managed on LCIG alone (Antonini et al. 2017). For example, expert consensus guidelines suggest that COMT-inhibitors are useful to reduce the required levodopa dose of LCIG, particularly when the daily dose exceeds 100 ml (2000 mg or 1 cassette) (Buhmann et al. 2017). Indeed, in the GLORIA study, 56.5% of patients included in the LCIG registry were also on a COMT inhibitor (Antonini et al. 2017). Such observations are in keeping with an early pilot study of LCIG given with oral entacapone or tolcapone, which showed that the total levodopa dose could be reduced by 20% without worsening of motor fluctuations (Nyholm et al. 2012). Similar reductions in total levodopa dose have been reported when LCIG is administered with opicapone (Leta et al. 2020). More recently, a small study in Japanese patients (*n* = 8) reported that an afternoon dose of entacapone was useful in ameliorating afternoon OFF symptoms in patients treated with LCIG (Miyaue et al. 2024).

A subcutaneous levodopa/carbidopa prodrug infusion (fos-levodopa-fos-carbidopa, commercialised as Produodopa^®^) has now also reached the market. Interestingly, although subcutaneous infusion has been developed to improve tolerability, *more* patients discontinued therapy over 12 weeks with fos-levodopa-fos-carbidopa than with LCIG (22% vs. 5%, respectively) (Soileau et al. 2022; Olanow et al. 2014). Such discrepancies might reflect the observation that patients with PD find ongoing skin issues (72% of patients had infusion site events reported as AEs with fos-levodopa-fos-carbidopa) intolerable when considered in the context of an easily reversible therapy. Fos-levodopa-fos-carbidopa has been developed as a complete replacement for levodopa-containing medications and COMT-inhibitors, and as such data on its combination with COMT inhibitors is scarce. However, it is noteworthy that patients treated with fos-levodopa-fos-carbidopa still experienced an average of ∼3 h of OFF time at the end of study (Soileau et al. 2022) compared with ∼2 h with LCIG (Olanow et al. 2014). The relative bioavailabilities of the two infusion methods remains to be compared.

## Putting two and two together: combining Levodopa infusion with COMT Inhibition

**Fig. 2:**
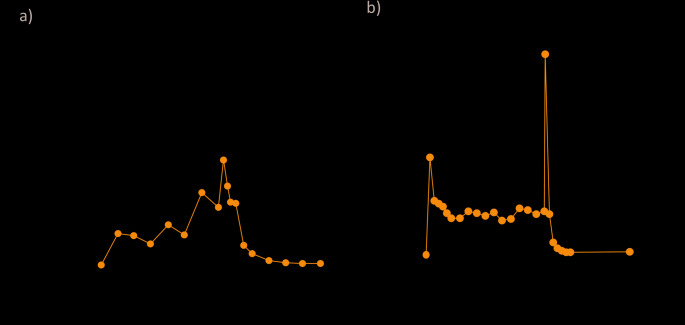
Entacapone plasma kinetics when given **a** orally and **b** as continuous infusion. **a** Day 1 pharmacokinetic mean entacapone plasma concentrations in healthy volunteers (*n* = 12) who received multiple doses of entacapone per day. Figure adapted from (Rouru et al. 1999) with permission; **b** Pharmacokinetic mean entacapone plasma concentrations in patients with Parkinson’s disease (*n* = 6) who received 14 h of dosing with LECIG at 80% of their usual LCIG dose. The peak at 14 h reflects flushing of the tube with water after stopping the infusion

The development of LECIG which combines levodopa, carbidopa and entacapone in one continuous intestinal gel infusion (LECIGON^®^) was the next logical step to optimise the effect of levodopa in advanced PD. In particular, continuous infusion of entacapone overcomes many of the deficiencies of the drug when used by standard oral therapy as listed earlier. The short plasma half-life is no longer a problem as entacapone is delivered to its site of absorption in the gastro-intestinal tract. The low oral bioavailability is no longer relevant as the drug is infused on a continuous basis and not as fragmented oral boluses. Steady state levels of entacapone are achieved by continuous infusion so that fluctuations in the extent of COMT inhibition do not occur in the same way as following standard oral administration (Fig. 2). Continuous infusion also helps overcome the heterogeneity of response caused by polymorphisms in the COMT gene. Concerns over effects on levodopa and carbidopa absorption are mitigated as all three agents are delivered on a continuous basis and continuous delivery maximises the extent to which COMT inhibition will occur (Kaakkola 2010). Inhibiting COMT-mediated conversion of levodopa into 3-OMD also has the theoretical advantage of reducing homocysteine production and thereby potentially reducing the risks of peripheral neuropathy, which has associated with levodopa infusion (Merola et al. 2016).

As with Stalevo, the approval of LECIG was based on the well-established pharmacology, efficacy, and safety of its components (both in oral and intestinal gel form) and supported by pharmacokinetic studies (Nyholm et al. 2012; Senek et al. 2017). LECIG was first approved by the Swedish medical products agency in 2018 (Swedish Medical Products Agency 2018) and followed by several European countries.

## Introducing levodopa/carbidopa/entacapone intestinal gel (LECIGON)

**Fig. 3:**
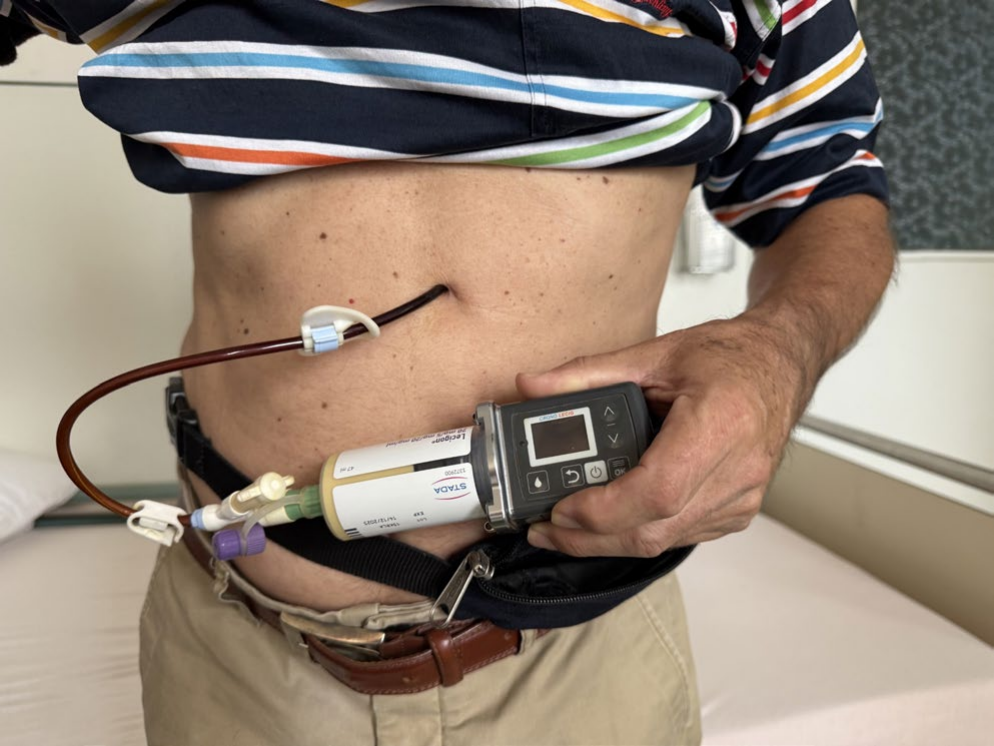
A Parkinson’s patient using LECIG infusion

Like LCIG, LECIG is continuously infused directly into the small intestine, bypassing the stomach and avoiding the pharmacokinetic problems associated with gastric emptying. LECIG is supplied in 47 ml cartridges each containing 940 mg levodopa (20 mg/ml), 235 mg carbidopa monohydrate (5 mg/ml) and 940 mg entacapone (20 mg/ml). It is delivered using the specially designed Crono^®^ LECIG pump that measures 55 × 150 mm and has a total weight of 227 g (the same weight as an iPhone 16 pro max) (Fig. 3). LECIG is adaptable to the patient’s condition, allowing adjustments in both continuous and bolus dosing. The device is programmed to provide three types of dosing:

1. Morning dose: An initial dose is often given at the start of the day to quickly bring levodopa levels up and manage early morning OFF.
2. Maintenance dosing: The maintenance rate is set to continuously deliver LECIG throughout the waking hours based on the individual needs of the patient. Three different flow rates can be provided; sometimes lower doses are used in the afternoon. A low nighttime maintenance dose may also be introduced to enable 24-hour dosing if required.
3. Bolus dosing: Bolus doses can be used ‘as needed’ for rapid relief of acute OFF episodes that can occur despite continuous maintenance dosing.

### Pharmacokinetic studies

**Fig. 4:**
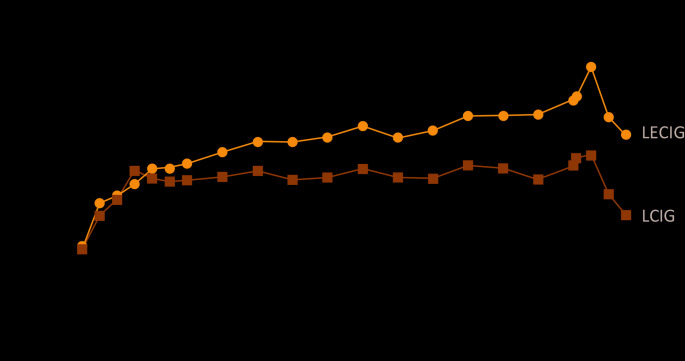
Mean levodopa plasma concentrations with LECIG vs. LCIG (Senek et al. 2017). Pharmacokinetic mean (± SE) dose-adjusted plasma concentrations of levodopa given as LCIG and LECIG (*n* = 11). Blood samples were drawn immediately prior to dosing, half-hourly between 0 and 3 h, and hourly between 3 and 14 h

The potential to manage patients with LECIG using lower levodopa doses (vs. LCIG) was explored in a randomised crossover study (Senek et al. 2017). In this study, 11 patients with advanced PD currently treated with LCIG were treated with LECIG given at a reduced levodopa dose (participants received LECIG morning doses corresponding to 80–90% of their individual morning dose of LCIG, 80% of the LCIG maintenance dose, and 80% of extra doses) over 14 h (Senek et al. 2017). Despite the 20% reduced levodopa dose with LECIG, systemic exposure for levodopa did not differ significantly between treatments (AUC_0 − 14_ ratio of 1.10 [95% CI, 0.95–1.17], *p* = 0.3) but the dose adjusted levodopa exposure was significantly higher with LECIG vs. LCIG (Fig. 4) (Senek et al. 2017). Maximal plasma concentrations (C_max_) were also similar (ratio of 1.12 [95% CI, 0.98–1.19], *p* = 0.09). Importantly, treatment response scale (TRS) scores assessing motor response showed no difference between the LECIG and LCIG treatment groups confirming that the therapeutic levodopa dose can be successfully reduced with LECIG without lowering levodopa exposure. As predicted, continuous infusion of entacapone resulted in a steady plasma entacapone profile (Fig. 2b). Of note, plasma 3-OMD levels increased by 22% when switching from LECIG to LCIG but decreased by 35% when switching from LCIG to LECIG (Senek et al. 2017).

Using these data, a population pharmacokinetic model was developed to better characterise the dose adjustment needed when switching patients from LCIG to LECIG (Senek et al. 2020). The model was used to simulate three alternative dose regimens for LECIG based on prior LCIG dosing (i) all the LCIG morning dose with no reduction in the continuous maintenance dose, (ii) a 20% lower morning dose with a 20% lower maintenance dose, and (iii) all the morning dose with a 35% lower maintenance dose. These data indicated that the 20% reduction in levodopa dose when initiating LECIG did not completely adjust for the COMT inhibition effect of entacapone and the continuous LECIG maintenance dose should be decreased by 35%, while the morning dose should be maintained at 100% (Senek et al. 2020). Continuous infusion of COMT inhibitors allows for greater dose reductions compared to oral adjunct therapy, due to improved bioavailability from direct delivery to the duodenum or jejunum. This route bypasses some of the metabolic losses associated with oral administration, resulting in more effective COMT inhibition. For instance, an estimated 6–11% of orally administered entacapone is lost through intestinal metabolism (Alqahtani and Kaddoumi 2015) and the gut also appears to be an important secondary site of opicapone metabolism (Loureiro et al. 2022).

All patients in the study were genotyped for rs4680, as well as the DDC genes rs921451, and rs3837091 (Senek et al. 2020). Results showed no clear trend observed in the apparent clearance of levodopa (CL/F) between the high, intermediate, and low COMT activity subgroups – indicating that all patients, irrespective of COMT rs4680 polymorphism, have a high reduction in levodopa clearance with an addition of simultaneously infused entacapone (Senek et al. 2020).

## Real-world data with LECIG

The first cohort of patients using LECIG has been closely followed by the department of Neurology at Uppsala University Hospital, Sweden. In this ongoing cohort study, 24 patients (11 females and 13 males) with advanced PD who had been prescribed LECIG were followed up according to usual clinical practice with an outpatient visit every 6 months. Half (*n* = 12) of patients had switched directly from LCIG infusion to LECIG (3 patients had previously trialled LCIG and discontinued) and provided a comparison of their experiences (Öthman et al. 2021). Two patients were treated with subthalamic DBS combined with LECIG, and one had previously removed a DBS system due to severe infection. Other patients switched from CSAI (*n* = 2) or levodopa/carbidopa micro tablets [LC-5 (Gretarsdottir et al. 2021)] (*n* = 3) to LECIG (Öthman et al. 2021).

Baseline characteristics were as expected for an advanced population. The median age when starting LECIG was 71.5 years and patients were a median of 15.5 years out from PD diagnosis. At one year post study start (median LECIG treatment duration of 305 days), the median daily levodopa dose had decreased from 1210 mg [range 435–2400 mg] at baseline to 1040 mg [370–2000 mg] upon start of LECIG, to 1080 mg [510–1822 mg] at end of the initial follow-up period (Öthman et al. 2021). Of the patients who had not used any kind of levodopa infusion before, most reported that that their symptom control had improved (7 of 10 reported improvements, 2 reported no change, and 1 reported worsening). By contrast, 5 of 11 patients who had switched from LCIG to LECIG reported no change (4 reported improvement and 2 reported worsening) in their symptoms. While there was no obvious symptomatic efficacy benefit to the switch, most patients reported that they thought the new [LECIG] pump was improved vs. LCIG both with respect to user-friendliness and to changing cassette/syringe, and all patients thought that the smaller size of the pump was an improvement on their prior LCIG therapy (Öthman et al. 2021).

These patients have now been followed for up to 4 years (median 3.6 years) (Öthman and Nyholm 2024). Of the 24 patients, five (21%) had discontinued LECIG because of side effects, mostly diarrhoea, eight (33%) had died (while still receiving LECIG) and 11 (46%) patients were still on LECIG after a median duration of 3.6 (3.1–4) years. Of those patients who had discontinued therapy, the median time on LECIG was 1.1 years. These patients generally had a higher LECIG dosage compared with those still on LECIG – perhaps indicating a more advanced stage of the disease as well as a higher risk for side effects. Of note, discontinuations due to side effects (diarrhoea [*n* = 3], hallucinations [*n* = 1]) generally occurred early (within the first 6 weeks of treatment) (Öthman and Nyholm 2024). Recent work has revealed that patients with PD can develop lymphocytic microscopic colitis (characterised by a history of chronic watery diarrhoea) can develop as an idiosyncratic hypersensitivity reaction to PD drugs including levodopa/carbidopa (Steel et al. 2022), and entacapone (Rodrigues et al. 2022). More work needs to be done to understand the mechanisms behind these reactions, which are also seen with nonsteroidal anti-inflammatory drugs, proton pump inhibitors and selective serotonin reuptake inhibitors (Bonderup et al. 2014). As such, experts using LECIG recommend testing each patient’s tolerability of oral entacapone before starting LECIG infusion treatment (Öthman and Nyholm 2024; Szatmári et al. 2024).

Real world observations from this first cohort have recently been supplemented by findings from the Swedish registry for Parkinson’s disease (ParkReg) (Öthman et al. 2025). Overall, 150 patients were identified as starting treatment with LECIG between 2019 and 2022. Of these a third had switched from another device aided therapy and two had concomitant DBS therapy. The median age at LECIG initiation was 73 years and the median duration of motor symptoms was 17 years. Reported complications were mainly related to PEG-J tube (19%) and stoma (11%). Interestingly, only one patient (1%) reported diarrhoea as an AE during this study. While this may be an underestimation (because AE reporting was not mandatory) it may also reflect the Swedish approach of testing tolerability to oral entacapone before initiating LECIG. Overall, 13.3% of 150 patients discontinued LECIG and 7.3% died while on LECIG – however the reasons for discontinuation were not consistently available (Öthman et al. 2025). A third of patients (*n* = 53) in the ParkReg study also wore the Parkinson KinetiGraph (PKG) for objective motor symptom (bradykinesia, dyskinesia, fluctuations, and tremors) measurement and results confirmed good symptom control with a statistically significant improvement in PKG assessed motor fluctuation scores. While clinically relevant improvements in disease-specific PDQ-8 summary index score were seen with LECIG they were not apparent on generic quality of life assessment (EQ-5D).

Real world evidence from countries outside of Sweden is now starting to filter through. Santos-García and colleagues reported their observations of 74 patients who were treated with LECIG in 21 centres across Spain (Santos-García et al. 2025). In this study, treatment with LECIG reduced OFF time from a baseline mean of 5.2–1.9 h at end of study (change of 3.3 h) and the authors highlighted that LECIG reduced OFF time – not only in the group of patients with a direct initiation but also in those patients who switched from LCIG to LECIG (Santos-García et al. 2025). The frequency of many disabling non-motor symptoms, including depression, anxiety, sleep problems, fatigue, and pain, also significantly decreased under LECIG treatment. Of note, fewer than 7% of patients dropped out of therapy after a mean follow-up of approximately 6 months and no cases of diarrhoea were detected (Santos-García et al. 2025).

Two reports have been published documenting the Romanian experience. Szatmári and colleagues described their experience treating 20 consecutive patients with LECIG at a single University teaching hospital (Szatmári et al. 2024). Unlike other cohorts where many patients were switching between device-aided therapies (LCIG or CSAI), patients in this cohort were new to continuous infusion. Treatment significantly reduced daily OFF time from a mean of 4.8 ± 0.9 h/day when on oral treatment regimens (often combining dopamine agonists, MAO-B inhibitors, and oral COMT inhibitors) to a mean of 1.4 ± 0.5 h per day with LECIG (*p* < 0.001) (Szatmári et al. 2024). A quarter of patients (*n* = 5) could gradually reduce and subsequently discontinue their concomitant therapy with dopamine agonists and MAO-B inhibitors. In those patients with peak dose dyskinesia, the duration of dyskinesia was significantly reduced from 3.3 ± 1.3 h/day to 1.2 ± 0.6 h/day. As would be expected with continuous infusion that bypasses the stomach, initiation of LECIG resolved all issues of delays to ON, no-ON, and sudden OFFs. Interestingly, 8 of the 10 patients who reported freezing at baseline, no longer had this complication at study end (Szatmári et al. 2024). At this experienced hospital, while 16 of 20 patients reported minor post-procedural pain, no patient presented any significant complication during the evaluation period.

In a larger retrospective analysis of 74 patients treated across 12 centres across Romania, Szász and colleagues similarly reported that LECIG treatment resulted in improved motor symptoms, significantly decreased daily OFF time (from 5.7 to 1.7 h/day), and reduced painful dyskinesias (Szász et al. 2024). Of note they reported that while 80% of patients still used some adjunctive oral therapies alongside LECIG infusion, 15 patients (20%) were able to achieve satisfactory motor control with LECIG monotherapy. The median infusion time for LECIG was 16.6 h per day, however five patients were able to implement LECIG monotherapy but over 24 h. The indications for the administration of 24-h infusion were severe nighttime akinesia and early morning akinesia (Szász et al. 2024).

Finally, in a study of 30 patients initiated onto LECIG in Finland, Viljaharju and colleagues reported that the levodopa equivalent daily dose (LEDD) rose significantly between baseline before LECIG and 6 months with treatment (1230 mg vs. 1570 mg, *P* = 0.001). In this study, about a quarter (26%) of patients discontinued the treatment during the first 6 months of treatment. One patient died from a cause unrelated to LECIG treatment. Two patients stopped treatment due to difficulties in finding a suitable infusion rate, and two others due to neuropsychiatric issues. Another patient, who initially struggled with finding an optimal infusion rate, developed rhabdomyolysis due to severe dyskinesia and subsequently discontinued the treatment. Among the patients who switched from LCIG to LECIG treatment, only one discontinued during the follow-up period due to neuropsychiatric problems, while the remaining five patients continued with LECIG (Viljaharju et al. 2024).

Alongside these smaller studies a prospective, observational European study - ELEGANCE (NCT05043103) is currently ongoing. Recruitment is complete and the study includes 312 patients treated with LECIG infusion as part of routine clinical practice across 16 countries (the decision to prescribe LECIG was to have been independent of the decision to include the patient in the study). Interim results from the first 167 patients with a complete year of follow-up data have been recently published (Weiss et al. 2025). So far, 1 year discontinuation rates are low (1.8%) and the data support sustained control of motor fluctuations through 12 months of treatment (OFF-time was significantly reduced by a mean of 3.5 h and MDS-UPDRS Part IV [motor complication] scores were significantly reduced by a mean of 4.2 points, both *p* < 0.0001). These improvements were translated into significantly improved motor experiences of daily living, sleep, and quality of life. Patient-reported satisfaction with the LECIG pump was high and there was a low rate of bothersome dyskinesia and neuropsychiatric complications; AEs were mostly related to the PEG-J system (Weiss et al. 2025).

## Summary and conclusions

Enzyme inhibition with DDC inhibitors, and to a lesser extent MAO-B inhibitors, is an integral and widely accepted part of the process for maximising the efficacy of orally administered levodopa in the treatment of both the early and late stages of PD and key to overcoming the otherwise short duration of effect of individual levodopa doses. In contrast, the use of COMT inhibitors to block the second major metabolic pathway of levodopa was introduced later. Despite their proven ability to increase levodopa bioavailability and improve clinical outcomes, COMT inhibitors are often reserved for managing motor fluctuations in later stages of PD. The most common approach involves oral entacapone, either as an adjunct to levodopa or in combination formulations like Stalevo. However, as discussed in this review, this method is suboptimal due to the poor oral bioavailability of entacapone and its short duration of action. Overall, COMT inhibition remains underutilised and undervalued in PD management.

The alternative strategy for managing late-stage PD using levodopa has been to employ continuous drug delivery using an advanced therapy such as LCIG. Again, this is routinely combined with the simultaneous delivery of carbidopa to inhibit DDC to increase levodopa delivery to brain. While reducing OFF periods, continuous intestinal levodopa infusion does not abolish OFF-time and the other major route of levodopa metabolism through COMT remains unchecked. Combining continuous levodopa delivery with continuous delivery of entacapone to maintain COMT inhibition is the next logical step as it both maximises the effectiveness of levodopa by improving its plasma pharmacokinetic profile while simultaneously overcoming many of the limitations of oral entacapone administration. The accumulating evidence clearly shows that LECIG reduces motor fluctuations, decreases levodopa dose compared to levodopa/carbidopa alone and can lead to an improvement in both motor and non-motor symptoms. It now needs to take its place in the algorithm for the treatment of fluctuating PD that is ineffectively treated using oral medication.

Because of the need for surgery, intestinal advanced therapies such as LECIG are often only utilised for more advanced PD patients and instead, continuous subcutaneous drug delivery is considered as less invasive. Certainly, the choice of delivery route needs a decision based on the needs of individual patients and all currently used advanced technologies are valuable additions to the therapeutic toolbox. However, subcutaneous therapies come with their own disadvantages. Subcutaneous infusion of apomorphine can be highly effective but may lead to skin irritation and nodule formation and the risk of the adverse events associated with dopamine agonist use (Bhidayasiri et al. 2016). The subcutaneous infusion of fos-levodopa-fos-carbidopa again leads to site irritation including infections (Soileau et al. 2022) and is limited by the amount of drug passively absorbed from a single site making dosage adjustment difficult. Additionally, the high and prolonged levodopa plasma concentrations, even at low infusion rates, of fos-levodopa-fos-carbidopa have also been linked to an increased risk of confusion and neuropsychiatric adverse events (Brohée et al. 2025). While intestinal therapies are invasive, they do not result in widespread inflammation and skin irritation, and they do not limit the amount of levodopa that can be absorbed because the capacity of the active transport from gut to blood is almost limitless. Combining continuous levodopa/carbidopa with entacapone solves many of the current problems associated with oral levodopa and entacapone use and the improvements in symptom control may be life changing. As real-world data on LECIG continue to accumulate, its role in managing motor fluctuations in patients with suboptimal oral control is likely to expand, and its potential for earlier use in the disease course warrants further exploration.
